# The Effectiveness of a 5% Retinoic Acid Peel Combined with Microdermabrasion for Facial Photoaging: A Randomized, Double-Blind, Placebo-Controlled Clinical Trial

**DOI:** 10.1155/2017/8516527

**Published:** 2017-02-15

**Authors:** Gita Faghihi, Saghi Fatemi-Tabaei, Bahareh Abtahi-Naeini, Amir Hossein Siadat, Giti Sadeghian, Mohammad Ali Nilforoushzadeh, Hamed Mohamadian-shoeili

**Affiliations:** ^1^Skin Diseases and Leishmaniasis Research Center, Department of Dermatology, Isfahan University of Medical Sciences, Isfahan, Iran; ^2^Cancer Research Center, Semnan University of Medical Sciences, Semnan, Iran; ^3^Skin Diseases and Leishmaniasis Research Center, Isfahan University of Medical Sciences, Isfahan, Iran; ^4^Skin and Stem Cell Research Center, Tehran University of Medical Sciences, Tehran, Iran; ^5^Department of Internal Medicine, Kermanshah University of Medical Science, Imam Reza Hospital, Kermanshah, Iran

## Abstract

*Background*. Tretinoin has been shown to improve photoaged skin. This study was designed to evaluate the efficacy and tolerability of a 5% retinoic acid peel combined with microdermabrasion for facial photoaging.* Materials and Methods*. Forty-five patients, aged 35–70, affected by moderate-to-severe photodamage were enrolled in this trial. All patients received 3 sessions of full facial microdermabrasion and 3 sessions of either 5% retinoic acid peel or placebo after the microdermabrasion. Efficacy was measured using the Glogau scale. Patients were assessed at 2 weeks and 1, 2, and 6 months after treatment initiation.* Results*. The mean ± SD age of participants was 49.55 ± 11.61 years, and the majorities (73.3%) were female. Between 1 month and 2 months, participants reported slight but statistically significant improvements for all parameters (*P* < 0.001). In terms of adverse effects, there were statistically significant differences reported between the 5% retinoic acid peel groups and the control group (*P* < 0.001). The majority of adverse effects reported in the study were described as mild and transient.* Conclusion*. This study demonstrated that 5% retinoic acid peel cream combined with microdermabrasion was safe and effective in the treatment of photoaging in the Iranian population. This trial is registered with IRCT2015121112782N8.

## 1. Introduction

Facial skin aging is characterized by wrinkles, laxity, roughness, lentigines, telangiectasia, and actinic keratosis [1–3].

Skin aging is influenced by several factors including genetics, environmental exposure (including Ultra-Violate (UV) radiation, xenobiotics, and mechanical stress), hormonal changes, and metabolic processes [4]. Solar UV radiation, via the process of photoaging, is the single most-common factor responsible for skin aging [5].

Many treatment options are available to control and reverse photoaging in facial skin [4]. Clinical studies as well as histological data suggest that tretinoin improves facial appearance following photodamage if used in the proper way [4].

In several double-blind, placebo-controlled studies, the efficacy of topical tretinoin was evaluated as a treatment for photoaging [6, 7].

Chemical peelings have also been found to be an effective treatment for moderate to severe photoaging [8].

Over the last few years, the number of clinical studies researching combination therapies to find more effective clinical treatments and cosmetic procedures for photoaging has increased [9].

Beneficial effects from microdermabrasion followed by a 5% retinoic acid chemical peel have been reported in a small study [10].

To the best of our knowledge, the long-term benefits of a combination of 5% retinoic acid peel with microdermabrasion for moderate to severe photoaging have not been studied in a randomized, double-blind, placebo-controlled study. Therefore, we investigated the efficacy and safety of a much higher concentration of topical 5% retinoic acid peel cream combined with microdermabrasion in patients with photoaging during a 6-month follow-up period after treatment initiation.

## 2. Materials and Methods

### 2.1. Participants

This was a randomized, comparative, split-face, evaluator-blinded clinical study. The Institutional Review Board of Isfahan University of Medical Sciences, Isfahan, Iran, approved the protocol of this study (Grant Number 394084). Written informed consent was obtained from each patient before enrollment.

The participants were comprised of 45 healthy Caucasian subjects with Fitzpatrick skin prototypes II and III, aged 35–70 years, who either attended or were referred to the dermatology clinics of Al-Zahra and Noor hospitals for the treatment of moderate to severe facial photoaging according to the Glogau classification (Table 1).

Patients were not included in the study if they had been pregnant, were breastfeeding, had any genodermatoses which may interfere with aging skin changes, showed premature aging, were using other concomitant treatments, had connective tissue disorders, were previously treated with oral retinoids in the six months prior to the study, used superficial chemical peels or microdermabrasion in the three months prior to the study, received medium or deep chemical peels or laser ablation within six months of the study, met the criteria for alcohol addiction, had infectious or inflammatory facial dermatoses, had photodermatosis, and did not need to avoid sun exposure.

### 2.2. Interventions

After cleaning the skin, all patients received a microdermabrasion treatment (30 cmHg, aluminum oxide crystals, Zagromed®) symmetrically across their entire face, without causing bleeding. After removing the crystals with 0.9% normal saline (0.9% sodium chloride, PLC, Tehran, Iran), a thin layer of each peel was applied a single time on each side of the face, with the control or intervention peel selected randomly. Faces were washed 4 hours after the application of the topical peel. Subjects dried their faces with paper towels. Then, they applied sunscreen.

Participants were asked to use broad-spectrum (UVA and UVB) sunscreen of SPF 50+ for 2 weeks prior to the initiation of the study [11].

After each peel session, proper use of this sunscreen every three hours for a minimum of 4 days during exposure to the sun was advised. Physical methods to minimize sun exposure, including hats and sunglasses, were discussed with each participant. The uses of cosmetics, including perfume, and the ingestion of photosensitizing agents were not allowed during the study.

The 5% retinoic acid peel was prepared at Isfahan University's Department of Pharmacy. The 5% retinoic acid peel was prepared from powder dissolved in ethanol and mixed with lubricant cream, to a 5% concentration. The placebo was prepared using a neutral cream as a control. Both creams (5% retinoic acid and placebo) were similar in appearance, odor, and color, and manufactured by the same pharmacist. Throughout the study, the person who administered the medication and the patients were blind to the peel cream assignments.

Each participant took part in three sessions of this combination therapy with a month-long interval between each session.

### 2.3. Outcome Assessment

Efficacy was measured using changes in participants' Glogau classifications. The results were assessed using standard photographs taken before and after each treatment. Each patient underwent photography in the same condition with a digital camera (Canon Power Shot G12; Canon Components Inc., Japan) carried out by one person. Patients were assessed by two blinded dermatologist evaluators at baseline and at 2, 4, 8, and 24 weeks after the start of the treatment. The presence of any possible participant-recorded side effects during intervals between each session was documented, along with any residual possible side effects assessed at each visit by a dermatologist.

### 2.4. Statistical Analysis

The data were analyzed using SPSS 16 software (SPSS, Chicago, IL, USA). To compare the reductions in Glogau classification scores at end of the treatment in relation to baseline, the Friedman test and Wilcoxon test were used. McNemar's test was employed to compare the baseline data and frequency of side effects between the conditions.

## 3. Results

A total of 45 participants were enrolled in the study, and all of them completed it. The mean ± SD age of participants was 49.55 ± 11.61 years and the majority of participants (73.3%) were female. More than half (57%) of the participants had a type III skin phototype. The demographic profile and basic data for patients are shown in Table 2.

**Figure 1:**
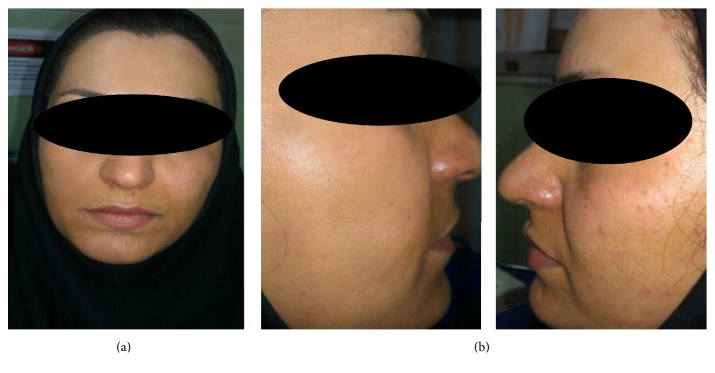
Clinical appearance of facial skin in a 37-year-old female with advanced photoaging at 6 months after (a) microdermabrasion and (b) 5% retinoic acid peel combined with microdermabrasion.

The results of the study demonstrated that when compared to the control, a 5% retinoic acid peel combined with microdermabrasion can result in clinically and statistically significant improvements in multiple aspects of facial photodamage (Figure 1). There was a statistically significant decrease in the mean ± SD Glogau classification score following the treatment among the 5% retinoic acid peel group (*P* < 0.001).

At baseline, 75.5% of the patients reported advanced to severe ratings for all skin parameters measured. Between weeks 4 and 8, subjects reported slight improvements that were statistically significant for all parameters (*P* < 0.001) (Table 3).

There was also an increase in the percentage of patients categorized as having a mild to moderate Glogau classification score after treatment.

At weeks 8 and 24, the 5% retinoic acid peel group showed statistically greater improvements compared to the control group (*P* < 0.001).  Table 4 shows the responder and nonresponder participants in both groups in terms of sex, skin type, and age (Table 4).

There were significant differences for both groups for the mean ± SD ages of participants, where responders were significantly younger in the microdermabrasion with 5% retinoic acid group (40.73 ± 8.79 years versus 53.97 ± 10.34 years; *P* < 0.001) and the microdermabrasion with placebo group (38.00 ± 6.16 years versus 53.76 ± 10.20 years; *P* < 0.001) (Table 4).

The most frequent side effects that occurred in the two groups were transient erythema in localized areas (*P* = 0.049). Sixteen (35.6%) and 13 (28.9%) patients reported erythema and scaling, respectively. Incidences of adverse events were significantly higher in the 5% retinoic acid peel group than in the control group.

Using McNemar's test for adverse effects, there was statistically significant difference in 5% retinoic acid peel group versus the control group (*P* < 0.001) (Table 5).

There was no significant difference in erythema by skin phototype in either groups (*P* > 0.05).

**Figure 2:**
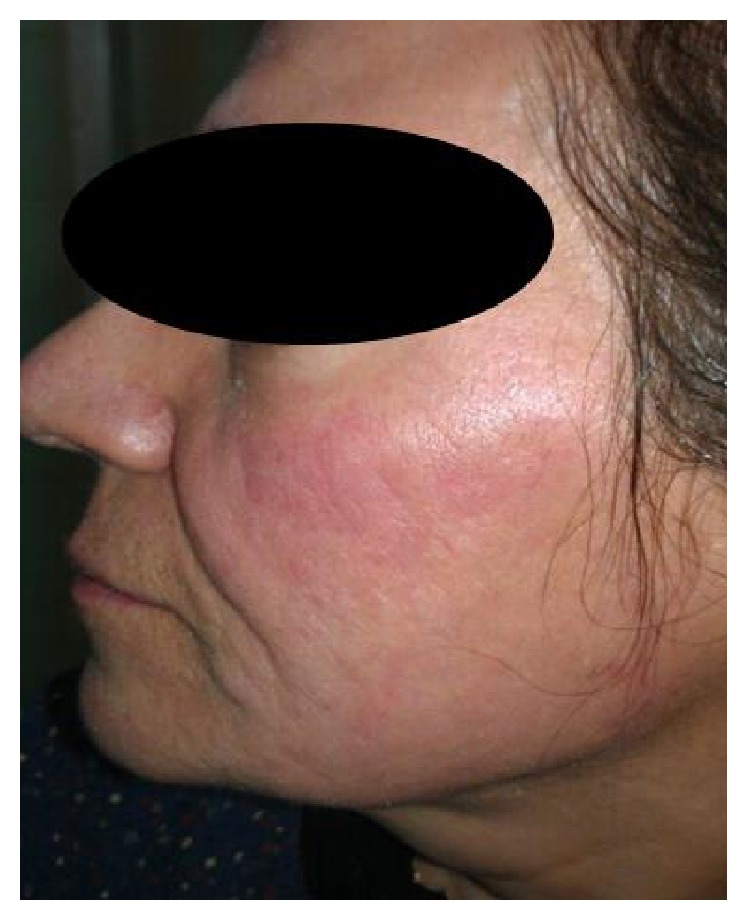
Erythema at the site of application 5% retinoic acid peel combined with microdermabrasion in a 35-year-old female. The erythema gradually faded after 3 weeks.

Most participants had postprocedural erythema, and a short course of a medium-potency topical corticosteroid was administrated for relief of symptoms. This transient complication resolved within two weeks for all patients (Figure 2). There were no cases of persistent erythema in our study. Other transient complications included burning sensations, mild scaling, and mild edema that gradually resolved during one week. None of the participants reported postinflammatory pigmentation or ulceration from erythema.

## 4. Discussion

This study demonstrated the efficacy of 5% retinoic acid peel cream combined with microdermabrasion as a treatment for facial skin photodamage.

Although tretinoin has been used in dermatology since the 1960s, its potential in the treatment of aging was realized no earlier than the 1980s [12]. Most 6-month studies have compared the efficacy of various low strengths of tretinoin to reach a concentration that is optimal for the treatment of photoaging [13].

These 6-month studies did show significant improvements in the clinical signs of photoaging but the improvements in skin condition continued even after 6 months [6, 14]. High-strength tretinoin treatment has been evaluated in the treatment of photoaging because the beneficial effects of conventional tretinoin therapy appear slowly and over a long period of time, which often leads to the discontinuation of therapy. Retinoid-related adverse effects include irritation, erythema, and dermatitis [15, 16].

Cucé et al. evaluated the clinical and histological efficacy and safety of tretinoin peeling procedures administered twice a week in concentrations of 1%–5% for photodamaged skin. Histological assessments carried out after 15 days showed compaction of the stratum corneum [17]. Although high-strength tretinoin has shown potential for treating photoaging, the existing studies have been carried out in small populations. Therefore, large-scale, multicentric studies using standard tretinoin therapy as a control are required to confirm its efficacy. Reviews of studies on combination treatments for photoaging demonstrated the superior clinical efficacy of multiple treatment modalities when used in combination [9].

A study by Hexsel et al. verified the clinical and histological parameters of patients who received a combination of microdermabrasion and 5% retinoic acid peel [10]. The results of their study showed that successive treatment with microdermabrasion and a 5% retinoic acid peel delivers greater improvements in cases of photodamage than retinoic acid peels applied alone. The more important limitation of that study was the small number of participants [10].

As with our participants, the participants in Hexsel et al. did not report side effects other than those previously reported in the literature [10].

In medium-depth and deep resurfacing or in combination resurfacing methods, transient erythema, flushing, increased skin temperature, pruritus, edema, milia formation, and mild alterations in mood are typical occurrences, so they should not be classified as true complications. Patient reassurance may be all that is necessary for these symptoms, since these problems usually resolve spontaneously. True complications that may develop within a treated area include infection, delayed wound healing, persistent erythema, scarring, and pigmentary or textural abnormalities.

By using microdermabrasion on the skin, topical therapeutics can be applied at the conclusion of the treatment, which will reach levels much deeper than when typically applied to the surface of the skin, as the skin barrier function of the stratum corneum is temporarily compromised [18]. Therefore, slightly greater improvements in the histological features of photoaging can be achieved with the combination of microdermabrasion followed by a 5% retinoic acid chemical peel versus a 5% retinoic acid chemical peel administered alone [10].

In our study, in both groups, younger subjects had a better improvement in responder group.

There is a suggestion that improvements in photoaged skin include elasticity and wrinkles after chemical peeling can be attributed to increase of collagen content [19, 20].

The overall collagen content per unit area of the skin surface has been known to decline with aging [21].

Given these issues, younger patients with higher collagen content per unit area of the skin surface will be expected to show better treatment responses.

There are several limitations in our study, including the duration of the follow-up, the lack of histopathological evidence to confirm treatment efficacy, and the small sample size. Our study had several limitations, but the importance of our study has been focused on observing the clinical effect of adding 5% retinoic acid peel to microdermabrasion, which had not been studied before in a randomized, double-blind, placebo-controlled clinical trial. Based on our results, the combination of a 5% retinoic acid peel combined with microdermabrasion may be a promising and effective technique for the treatment of facial photoaging.

Additional research into the efficacy and mechanisms of action of combined therapies for photoaging is needed to improve the science and clinical practice of the treating photoaging.

## 5. Conclusions

The combination regimen used in this study was shown to be safe and effective for the treatment of moderate to severe photoaging at a follow-up of 24 weeks after treatment initiation. Additional clinical studies with larger populations and more objective methods for clinical evaluation would further reveal the efficacy of this combination treatment for moderate to severe photoaging.

## Figures and Tables

**Table 1:** Glogau classification of photoaging.

Group	Classification	Typical age	Description	Skin characteristics
I	Mild	28–35	No wrinkles	Early photoaging: mild pigment changes, no keratosis, minimal wrinkles, minimal or no makeup
II	Moderate	35–50	Wrinkles in motion	Early to moderate photoaging: early brown spots visible, keratosis palpable but not visible, parallel smile lines begin to appear, wears some foundation
III	Advanced	50–65	Wrinkles at rest	Advanced photoaging: obvious discolorations, visible capillaries (telangiectasia), visible keratosis, wears heavier foundation always
IV	Severe	60–75	Only wrinkles	Severe photoaging: yellow-gray skin color, prior skin malignancies, wrinkles throughout: no normal skin, cannot wear makeup because it cakes and cracks

**Table 2:** Demographic profile and basic data of patients with photoaging.

Age (years)	
Range	35–70
Mean (±SD)	49.55 ± 11.61
Sex	
Male	12 (26.7%)
Female	33 (73.3%)
Skin phototype	
Type II	19 (42.2%)
Type III	26 (57.8%)
Glogau classification	
Mild	0 (0.00%)
Moderate	11 (24.5%)
Advanced	24 (53.3 %)
Severe	10 (22.2%)

**Table 3:** 

Grouping	Glogau classification	Period of study					*P* value (Friedman test)
		Base	After 2 weeks	After 4 weeks	After 8 weeks	After 24 weeks	
Microdermabrasion with tretinoin	Mild	0 (0.00%)	0 (0.00%)	1 (2.2%)	2 (4.4%)	6 (13.3%)	<0.001
	Moderate	11 (24.5%)	11 (24.5%)	10 (22.3%)	13 (28.9%)	6 (13.3%)	
	Advanced	24 (53.3%)	24 (53.3%)	24 (53.3%)	21 (46.7%)	23 (51.2%)	
	Severe	10 (22.2%)	10 (22.2%)	10 (22.2%)	9 (20%)	10 (22.2%)	

Microdermabrasion with placebo	Mild	0 (0.00%)	0 (0.00%)	1 (2.2%)	1 (2.2%)	2 (4.4%)	0.109
	Moderate	11 (24.5%)	11 (24.5%)	10 (22.3%)	11 (24.5%)	9 (20%)	
	Advanced	24 (53.3%)	24 (53.3%)	24 (53.3%)	24 (53.3%)	24 (53.3%)	
	Severe	10 (22.2%)	10 (22.2%)	10 (22.2%)	9 (20.0%)	10 (22.2%)	

*P* value (Wilcoxon test)		1	1	1	0.045	0.025	

**Table 4:** Compression of responder and nonresponder participants in both groups by sex, skin phototype, and age.

Characteristic		Microdermabrasion with tretinoin			Microdermabrasion with placebo		
		Responder (15)	Nonresponder (30)	*P* value	Responder (12)	Nonresponder (33)	*P* value
Sex (*n*%)	Male (12)	1 (8.3%)	11 (91.7%)	0.03	0 (0%)	12 (100%)	0.02
	Female (33)	14 (42.4%)	19 (57.6%)		12 (36.4%)	21 (63.6%)	

Skin type (*n*%)	Type II (19)	7 (36.8%)	12 (63.2%)	0.75	7 (36.8%)	12 (63.2%)	0.30
	Type III (26)	8 (30.8%)	18 (69.2%)		5 (19.2%)	21 (80.8%)	

Age (Mean ± SD)		40.73 ± 8.79	53.97 ± 10.34	<0.001	38.00 ± 6.16	53.76 ± 10.20	<0.001

**Table 5:** Frequent adverse events reported in the study following treatment in intervention or control conditions.

Adverse events	Microdermabrasion with tretinoin (*n*%)	Microdermabrasion with placebo (*n*%)	*P* value
Pruritus	9 (20%)	2 (4.4%)	0.02
Erythema	16 (35.6%)	9 (20%)	0.049
Scaling	13 (28.9%)	6 (13.3%)	0.036
Burning sensation	7 (15.6%)	0	0.006

## References

[1] Fisher G. J., Kang S., Varani J. (2002). Mechanisms of photoaging and chronological skin aging. *Archives of Dermatology*.

[2] Samuel M., Brooke R. C., Hollis S., Griffiths C. E. (2005). Interventions for photodamaged skin. *The Cochrane Database of Systematic Reviews*.

[3] Makrantonaki E., Zouboulis C. C. (2007). William J. Cunliffe Scientific Awards. Characteristics and pathomechanisms of endogenously aged skin. *Dermatology*.

[4] Poon F., Kang S., Chien A. L. (2015). Mechanisms and treatments of photoaging. *Photodermatology, Photoimmunology & Photomedicine*.

[5] Rittié L., Fisher G. J. (2002). UV-light-induced signal cascades and skin aging. *Ageing Research Reviews*.

[6] Bagatin E., Guadanhim L. R. S., Enokihara M. M. S. S. (2014). Low-dose oral isotretinoin versus topical retinoic acid for photoaging: A Randomized, Comparative Study. *International Journal of Dermatology*.

[7] Randhawa M., Rossetti D., Leyden J. J. (2015). One-year topical stabilized retinol treatment improves photodamaged skin in a double-blind, vehicle-controlled trial. *Journal of Drugs in Dermatology*.

[8] Rendon M. I., Berson D. S., Cohen J. L., Roberts W. E., Starker I., Wang B. (2010). Evidence and considerations in the application of chemical peels in skin disorders and aesthetic resurfacing. *Journal of Clinical and Aesthetic Dermatology*.

[9] Tierney E. P., Hanke C. W. (2010). Recent advances in combination treatments for photoaging: review of the literature. *Dermatologic Surgery*.

[10] Hexsel D., Mazzuco R., Dal'Forno T., Zechmeister D. (2005). Microdermabrasion followed by a 5% retinoid acid chemical peel vs. a 5% retinoid acid chemical peel for the treatment of photoaging—A Pilot Study. *Journal of Cosmetic Dermatology*.

[11] Erbagci Z., Akcali C. (2000). Biweekly serial glycolic acid peels vs. long-term daily use of topical low-strength glycolic acid in the treatment of atrophic acne scars. *International Journal of Dermatology*.

[12] Mukherjee S., Date A., Patravale V., Korting H. C., Roeder A., Weindl G. (2006). Retinoids in the treatment of skin aging: an overview of clinical efficacy and safety. *Clinical Interventions in Aging*.

[13] Lever L., Kumar P., Marks R. (1990). Topical retinoic acid for treatment of solar damage. *British Journal of Dermatology*.

[14] Bouloc A., Vergnanini A. L., Issa M. C. (2015). A double-blind randomized study comparing the association of Retinol and LR2412 with tretinoin 0.025% in photoaged skin. *Journal of Cosmetic Dermatology*.

[15] Kligman D. E., Sadiq I., Pagnoni A., Stoudemayer T., Kligman A. M. (1998). High-strength tretinoin: a method for rapid retinization of facial skin. *Journal of the American Academy of Dermatology*.

[16] Kligman D. E., Draelos Z. D. (2004). High-strength tretinoin for rapid retinization of photoaged facial skin. *Dermatologic Surgery*.

[17] Cucé L. C., Bertino M. C. M., Scattone L., Birkenhauer M. C. (2001). Tretinoin peeling. *Dermatologic Surgery*.

[18] Karimipour D. J., Karimipour G., Orringer J. S. (2010). Microdermabrasion: an evidence-based review. *Plastic and Reconstructive Surgery*.

[19] Butler P. E. M., Gonzalez S., Randolph M. A., Kim J., Kollias N., Yaremchuk M. J. (2001). Quantitative and qualitative effects of chemical peeling on photo-aged skin: an experimental study. *Plastic and Reconstructive Surgery*.

[20] El-Domyati M. B. M., Attia S. K., Saleh F. Y., Ahmad H. M., Uitto J. J. (2004). Trichloroacetic acid peeling versus dermabrasion: a histometric, immunohistochemical, and ultrastrustural comparison. *Dermatologic Surgery*.

[21] Shuster S., Black M. M., McVitie E. (1975). The influence of age and sex on skin thickness, skin collagen and density. *British Journal of Dermatology*.

